# Validating physician-certified verbal autopsy and probabilistic modeling (InterVA) approaches to verbal autopsy interpretation using hospital causes of adult deaths

**DOI:** 10.1186/1478-7954-9-49

**Published:** 2011-08-05

**Authors:** Evasius Bauni, Carolyne Ndila, George Mochamah, Gideon Nyutu, Lena Matata, Charles Ondieki, Barbara Mambo, Maureen Mutinda, Benjamin Tsofa, Eric Maitha, Anthony Etyang, Thomas N Williams

**Affiliations:** 1 Department of Epidemiology and Demography, KEMRI-Wellcome Trust Research Programme, PO Box 230 Kilifi 80108, Kenya; 2 Nuffield Department of Medicine, John Radcliffe Hospital, Oxford OX39DS, UK; 3 Department of Paediatrics, John Radcliffe Hospital, Oxford OX39DS, UK; 4 Kilifi District Hospital, PO Box 9 Kilifi 80108, Kenya; 5 INDEPTH Network of Demographic Surveillance Sites, Accra, Ghana

**Keywords:** verbal autopsy, InterVA, validation, cause-specific mortality fraction, kappa, ROC

## Abstract

**Background:**

The most common method for determining cause of death is certification by physicians based either on available medical records, or where such data are not available, through verbal autopsy (VA). The physician-certification approach is costly and inconvenient; however, recent work shows the potential of a computer-based probabilistic model (InterVA) to interpret verbal autopsy data in a more convenient, consistent, and rapid way. In this study we validate separately both physician-certified verbal autopsy (PCVA) and the InterVA probabilistic model against hospital cause of death (HCOD) in adults dying in a district hospital on the coast of Kenya.

**Methods:**

Between March 2007 and June 2010, VA interviews were conducted for 145 adult deaths that occurred at Kilifi District Hospital. The VA data were reviewed by a physician and the cause of death established. A range of indicators (including age, gender, physical signs and symptoms, pregnancy status, medical history, and the circumstances of death) from the VA forms were included in the InterVA for interpretation. Cause-specific mortality fractions (CSMF), Cohen's kappa (κ) statistic, receiver operating characteristic (ROC) curves, sensitivity, specificity, and positive predictive values were applied to compare agreement between PCVA, InterVA, and HCOD.

**Results:**

HCOD, InterVA, and PCVA yielded the same top five underlying causes of adult deaths. The InterVA overestimated tuberculosis as a cause of death compared to the HCOD. On the other hand, PCVA overestimated diabetes. Overall, CSMF for the five major cause groups by the InterVA, PCVA, and HCOD were 70%, 65%, and 60%, respectively. PCVA versus HCOD yielded a higher kappa value (κ = 0.52, 95% confidence interval [CI]: 0.48, 0.54) than the InterVA versus HCOD which yielded a kappa (κ) value of 0.32 (95% CI: 0.30, 0.38). Overall, (κ) agreement across the three methods was 0.41 (95% CI: 0.37, 0.48). The areas under the ROC curves were 0.82 for InterVA and 0.88 for PCVA. The observed sensitivities and specificities across the five major causes of death varied from 43% to 100% and 87% to 99%, respectively, for the InterVA/PCVA against the HCOD.

**Conclusion:**

Both the InterVA and PCVA compared well with the HCOD at a population level and determined the top five underlying causes of death in the rural community of Kilifi. We hope that our study, albeit small, provides new and useful data that will stimulate further definitive work on methods of interpreting VA data.

## Background

Vital registration data in developing countries are incomplete and capture few physician-certified deaths [1]. Nevertheless, any meaningful health intervention policy or program must be informed by the causes of illness and death that are of greatest importance locally. Verbal autopsy (VA)-the interviewing of family members or caregivers about the circumstances of death after the event-offers one approach to the supplementation of this scarce but useful information. The government of Kenya suggested that the Kilifi, Nairobi, and Kisumu Demographic Surveillance System (DSS) sites use this approach to supplement national cause of death data. To allow data comparability, the latest version of the World Health Organization (WHO) Sample Vital Registration with Verbal Autopsy (SAVVY) tools were recommended for the sites [2].

The Kilifi Health Demographic Surveillance System (KHDSS) covers an area of 900 km^2 ^and a resident population of 250,000. Approximately 80% of patients admitted to Kilifi District Hospital (KDH) reside in this area. The population register is updated through re-enumeration rounds conducted every 3 to 4 months, and 1200 to 1500 deaths within the resident population are identified every year. More than 60% of these deaths occur outside the hospital where the causes of death are rarely recorded. Through collaboration with the Ministry of Health (MOH) at a local level, the KHDSS started collecting verbal autopsy data in 2008 with a view to establishing the underlying causes of death for the majority who die at home. Key sensitization messages were jointly developed and passed on to the community by staff working for both the KHDSS and the KDH. VA sensitization has subsequently become a routine process at the KDH and its surrounding health facilities.

The Kilifi integrated data managing system (KIDMS) is a computer-based system that links the KHDSS, pediatric, adult, and maternity ward surveillance systems in real-time through unique personal identifiers (PIDs). Deaths captured through any of these surveillance systems were captured in a single database and classified as neonates (0 to 27 days old), children (28 days to 14 years old) or adolescents and adults (15+ years old). The system generated the corresponding VA instruments and homestead maps for field interviews. Completed VA forms were edited, and the data were entered into a computer database for subsequent coding by a physician.

The main aim of the current study was to compare, at the population level, the distribution of underlying causes of adult deaths that are ascribed to a short list of 35 of the most common causes of death when using physician-certified verbal autopsy (PCVA) and the probabilistic InterVA model that are commonly used to interpret VA data with the distribution ascribed on the basis of physician diagnosis in a hospital, which we treat as our "gold standard."

## Materials and methods

### Study area and population

The KHDSS, first established in October 2000, serves as a framework for population-based epidemiological studies of diseases of local importance, monitors mortality trends, and is used to evaluate the impact of interventions of national public health importance. The area was initially mapped and all homesteads plotted using Garmin eTrex Venture^® ^hand-held geographical positioning system (GPS) units with an accuracy of three meters. The resident population was enumerated and individual details of age, sex, ethnicity, location/sub-location, and sleeping building unit (BU) of residence were recorded. Thereafter, births, deaths, in-migration and out-migration events, pregnancies, and new or demolished BU's were updated through census rounds conducted approximately three times a year. Cause of death data have been explored using the latest version of the WHO SAVVY tools since 2008.

**Figure 1:**
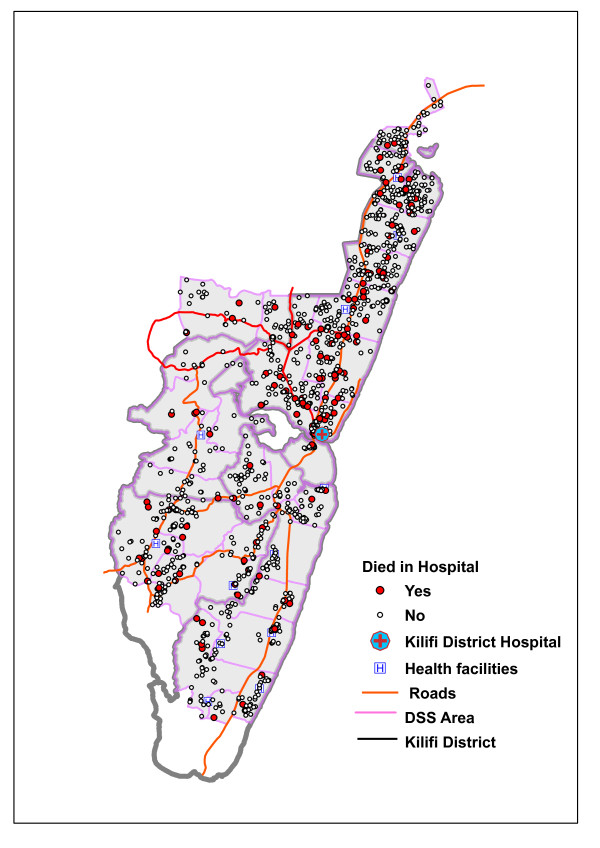
**The distribution of adult deaths, March 2007-June 2010**. The figure shows distribution of deaths used in this study. The red dots represent the 145 deaths that occurred in the Kilifi district hospital, and the white dots represent the overall death distribution between March 2007 and June 2010. The KHDSS area covers almost the entire district of Kilifi, making it sensible to generalize the results for community members living in the district.

The distribution of the adult deaths included in this study, which compares closely with the overall distribution of deaths from March 2007 to June 2010, is shown in Figure 1. The KHDSS area covers almost the whole of Kilifi district, making it possible to generalize the results of this study to the community living within the district.

### WHO SAVVY tools

The WHO SAVVY tools include three verbal autopsy questionnaires that are used to collect data on neonates (0 to 27 days old), children (28 days to 14 years old), and adolescents or adults (15+ years). Each questionnaire includes a short open narrative section followed by a series of closed questions. The narrative briefly explains the circumstances of death, while the closed questions provide details of specific signs, symptoms, and conditions. Introduction of the VA tools was preceded by a number of focus group discussions with community members to identify appropriate local terms for physical signs and symptoms and translate the forms into the local languages Giriama and Kiswahili. These translations were validated by back-translation by two independent teams of translators. Each interview took roughly 30 to 45 minutes to administer, 5 to 10 minutes to edit, and 5 to 10 minutes to enter into the computer.

### Physician certification of VA questionnaires

A computer-based work management system (written in FileMaker Pro™ V9.0; FileMaker, USA) was developed to capture deaths from the KIDMS, calculate age, print the corresponding VA instrument and homestead map, and provide data entry and coding screens. Physicians trained in the use of the WHO 10^th ^revision of the International Classification of Diseases (ICD-10) list [3] independently logged into the coding screen to review the VA questionnaires and determined both the immediate and the underlying cause of death. Using PID numbers for residents of KHDSS, the system compared the results of the two physicians to ascertain the cause of death in cases where there was agreement, identified disagreements for consensus, and coded the underlying cause of death according to the core three character code, as recommended by the ICD-10 [3]. On average, each review took 15 to 20 minutes.

### The probabilistic InterVA model

The InterVA (Interpreting Verbal Autopsy) model is a probabilistic model based on Bayes' theorem that can be used to determine the cause of death for each case by processing successive indicators to generate up to three likely causes of death for each case. The model was developed using an expert panel and was deliberately designed to be generic and not context dependent and to produce relatively broad cause of death categories. The development and details of the InterVA model have been described in detail previously [4,5]. The model is freely available in the public domain http://www.interva.net/. We recategorized our data to compare with the InterVA sublist of 35 causes of death. The input data for the model include signs, symptoms, medical history, and circumstances (injury, drowning, and accident) derived from the closed questions of the VA questionnaires. Adaptations made to the data to fit the model included compiling the same VA data into an input file for the InterVA model and processing it into cause of death data. The model also expects an input of ''high'' or ''low'' to reflect the local prevalence of two specific causes that often vary by more than an order of magnitude between settings: HIV and malaria, which in this study were set to ''high'' and ''high,'' respectively. Data on some InterVA indicators were not available in the WHO verbal autopsy tool and so remained null (see Additional file 1). It is also worth noting that the InterVA batch file was incompatible with recent versions of Microsoft Office™ (i.e., 2003/2007 or above), so we had to save our batch MS Excel file to a lower version. Data were transformed using both STATA Version 11 (Timberlake, USA) and SAS^® ^9.2 (SAS Institute, Inc.) software.

### Hospital cause of death: the gold standard

The cause of death at KDH (HCOD) was determined on the basis of high-quality clinical and laboratory data. The KEMRI-Wellcome Trust Research laboratories supporting the Kilifi District Hospital are Good Clinical Laboratory Practice (GCLP) accredited and are audited by international regulatory bodies on an annual basis. Patients admitted to KDH were examined according to a fixed protocol and samples were collected for malaria microscopy, hematology, and bacteriology. Other assays were performed as indicated by the clinical presentation of the patient. For those who died, the cause of death was determined by considering all the available evidence. The clinical data were captured online in real time using a standard questionnaire completed by the physician during the course of admission. The final diagnosis at death was selected from a modified, in-built ICD-10 list that included 590 diagnoses. For the purposes of this study, the hospital diagnosis that was based on standard guidelines (full medical history) and reflected the best judgment of the attending physician, substantiated by relevant radiological or laboratory investigations, was used as the gold standard.

### Ethical approval

The study was approved by the KEMRI/Wellcome Trust Kilifi - Scientific Coordinating Committee (SCC), the KEMRI Scientific Steering Committee (SSC), and the KEMRI/National Ethical Review Committee (ERC) in Nairobi. Community sensitization was conducted both by the Ministry of Health and the local community leaders. In addition, interviewers obtained informed consent from appropriate respondents.

### Data management and statistical analysis

We used HCOD as the gold standard for validating both PCVA and the InterVA model. Although the HCOD could be attributed to a maximum of two causes, we only considered the primary cause of death for the purposes of this comparative study. Where more than one cause was given, we selected the underlying cause of death (UCOD) as our unit of comparison. While the model is based on experts' opinion, the PCVA and HCOD are based on the ICD-10 guidelines. To enable comparisons in the context of a wide range of causes of death from the three methods, we first had to recode the data (see Additional file 2). Diagnoses that were included in all three methods (such as malaria, meningitis, and tuberculosis) retained their initial codes while lower-frequency diagnoses were recoded according to the more restricted range of classifications included in the InterVA model. For example, deaths attributed to "asthma" or "bronchitis" by HCOD or PCVA were recoded as "chronic respiratory diseases," while "rabies" and "tetanus" were recoded under "other acute infections." Similarly, causes such as "stroke," "hypertension," and "all heart conditions" were recoded as "cardiovascular diseases."

In situations in which there was no direct correlation, we had to recategorize the causes of death into broader cause groups. For instance, the InterVA model has two broad categories of bloody and nonbloody diarrhea for classifying all cases of diarrheal diseases. However, despite lack of microbiological evidence in verbal autopsy, the PCVA coded causes such as shigellosis and gastroenteritis. Such causes were therefore recoded into one broad category of diarrhea/gastroenteritis for comparison.

Another category "other acute infections" had conditions with fewer symptoms and/or nonspecific criteria to arriving at a particular diagnosis, and mostly termed as septicemia. The model did not distinguish pneumonia from sepsis and hence categorized both as a single COD of pneumonia/sepsis, but the physicians coded them separately. Pneumonia/sepsis was retained as a broad category and sepsis only was recategorized as "other acute infection."

While physicians could distinguish tuberculosis (TB) from HIV using the ICD-10 list, the InterVA model assigns TB and HIV as separate entities, making direct comparisons difficult in situations where TB and HIV occur together. TB cases reported in this current study, therefore, were cases that the physicians diagnosed as TB only.

**Figure 2:**
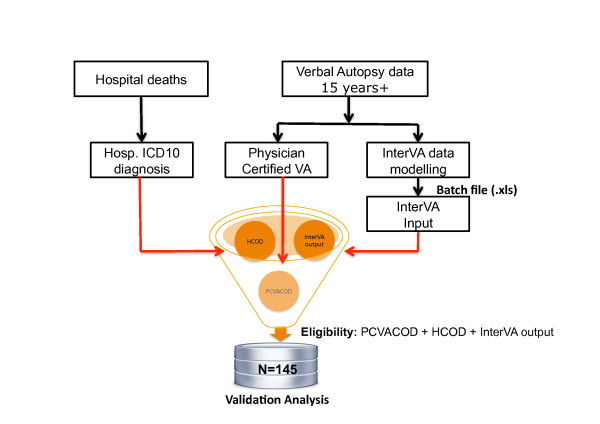
**Selection of adult deaths for inclusion in study conducted to validate both physician-certified verbal autopsy (PCVA) and the InterVA model against the hospital cause of death (HCOD)**. The figure shows the validation study design and the selection process of the adult deaths. The underlying cause of death determined by both InterVA and PCVA were compared against the corresponding HCOD.

The main causes of death determined by both InterVA and PCVA were compared against the corresponding HCOD (see Figure 2). Agreement was recorded as "1" where two or three methods agreed and "0" for no agreement. Cause-specific mortality fractions (CSMF) were used to measure agreement at population level and receiver operator characteristics (ROC) curve [6] was used to measure overall diagnostic performance of the methods. Case-by-case agreement between the methods was measured by Cohen's kappa (κ) statistic [7], sensitivity, specificity, and positive predictive values.

### Cause-specific mortality fractions

Cause-specific mortality fractions (CSMF) were determined as the proportion of all deaths that were attributable to a specific cause across the HCOD, the InterVA model, and the PCVA.

### Cohen's Kappa statistics (κ)

We used Cohen's kappa statistic (κ) to measure the level of agreement between the InterVA model or PCVA and the HCOD (the gold standard) for the underlying causes of death.

The kappa measure of agreement was stated as:

### Equation 1: Kappa measure of agreement

Where *P(A) *was the proportion of times the raters agreed, and *P(E) *was the proportion of times the raters were expected to agree by chance alone. Complete agreement corresponds to a κ value of 1, complete disagreement (i.e., purely random coincidences of rates) corresponds to a κ value of 0. A negative value of kappa would mean negative agreement. We used the following kappa (κ) scale to rate the strength of agreement as described previously [8]: a κ < 0.21 was considered poor, a κ between 0.21 and 0.40 fair, a κ between 0.41 and 0.60 moderate, a κ between 0.61 and 0.80 good, and a κ > 0.80 very good.

### Receiver operator characteristics (ROC) Curve

The area under the receiver operator characteristics (ROC) curve was calculated to measure the overall diagnostic performance (correctly diagnosing all the diseases) for both PCVA and InterVA against HCOD. For a method to be highly sensitive and specific, the area under the curve (AUC) should be close to one. The closer the curve follows the left-hand border and the top border of the ROC space, the more accurate the method. We considered the performance of our methods to be adequate if the area under the ROC curve exceeded 0.75

### Validity measures: sensitivity and specificity

Sensitivity, specificity, positive predictive value (PPV), and negative predictive value (NPV) with their 95% confidence intervals (CI) for the top five underlying causes of death were computed for PCVA and the InterVA model against the HCOD. The formulas for this calculation were defined as:

Where: TP = true positive, FP = false positive, TN = true negative, FN = false negative

We considered validity of a method to be adequate if the sensitivity and specificity exceeded 60% and 85%, respectively.

All analyses were carried out using R version 2.12.0 http://www.r-project.org/.

## Results

The KHDSS recorded 438 adult deaths which occurred in a hospital between March 2007 and June 2010. The current study included only those deaths (145) that occurred in the hospital (and their VA data coded by a physician). Deaths not meeting these criteria were dropped from the analysis. The mean age at death was 55 years (standard deviation 20 years), and 81 (56%) were males and 64 (44%) were females. The 145 deaths were successfully compared with the PCVA and the InterVA model. Ninety-one cases (63%) had two medically confirmed causes of death, giving a total of 236 HCOD. In the InterVA model output, 118 cases (81%) were assigned a single cause of death, 20 cases (14%) were assigned two causes of death, 2 cases (1%) were assigned three causes of death, and 5 cases (4%) were assigned as indeterminate. When the most possible cause of death assigned by the model disagreed with the HCOD, we considered both second and third likely causes of death, although such cases were few (only eight cases). On the basis of PCVA, a single cause of death was assigned in 143 (99%) cases, and 2 (1%) cases were coded as indeterminate.

The top five causes of death, which accounted for more than 60% of all deaths determined by the three methods, were HIV/AIDS-related, tuberculosis (pulmonary), meningitis, cardiovascular diseases, and diabetes. The InterVA model over reported tuberculosis as a cause of death compared to the other two methods, while PCVA overestimated diabetes.

**Figure 3:**
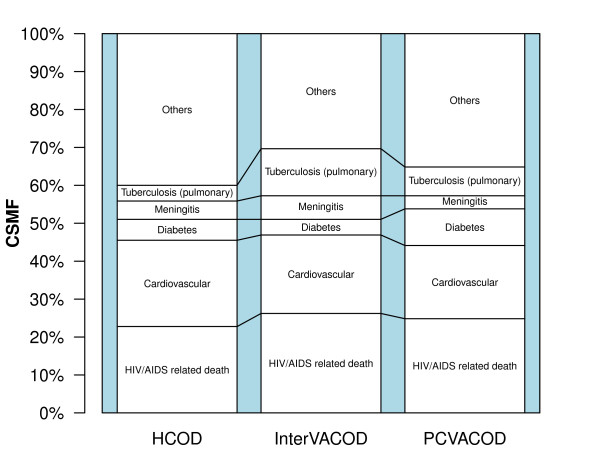
**Cause-specific mortality fractions for 145 adult deaths**. The figure shows cause-specific mortality fractions for 145 deaths derived from hospital causes of death, verbal autopsies interpreted by physician, and by the InterVA model. The CSMFs obtained were within ± 5% of those derived using the gold standard for the four most common causes of death (HIV-related, cardiovascular diseases, meningitis, and diabetes) and were within ± 8% of the gold standard value for tuberculosis (pulmonary).

The CSMFs obtained using the InterVA model and PCVA were compared separately with those obtained from the HCOD (Figure 3). The CSMFs obtained were within ± 5% of those derived using the gold standard for the four most common causes of death (HIV-related, cardiovascular diseases, meningitis, and diabetes) and were within ± 8% of the gold standard value for tuberculosis (pulmonary). The InterVA model attributed 38/145 (26.2%) deaths to HIV/AIDS, whereas the physicians and the HCOD attributed 36/145 (24.8%) and 33/145 deaths (22.7%), respectively. The InterVA model, PCVA, and HCOD all estimated similar CSMFs for cardiovascular diseases. On the other hand, PCVA attributed 14 (9.6%) deaths to diabetes, while the InterVA model and HCOD attributed 6 (4.1%) deaths and 8 deaths (5.5%), respectively. Furthermore, the InterVA model assigned three times as many deaths to tuberculosis (pulmonary) as HCOD. The InterVA model, PCVA, and HCOD attributed 9 (6.2%), 5 (3.4%), and 7 (4.8%) deaths respectively to meningitis.

The Kappa (κ) indicators for method agreement are shown in Table 1. The overall multirater kappa value across all three methods was 0.41 (95% CI: 0.37, 0.48), with agreement being better for females (κ = 0.48, 95% CI: 0.44, 0.52) than for males (κ = 0.35, 95% CI: 0.32, 0.38). Agreement between each method and the gold standard was fairly good (most κ > 0.40). PCVA versus HCOD yielded a higher kappa value (κ = 0.52, 95% CI: 0.48, 0.54), while InterVA versus HCOD yielded a kappa (κ) value of 0.32 (95% CI: 0.30, 0.38).

**Table 1:** Kappa (**κ**) statistics for agreement of the three methods among the 145 adult deaths

Methods	κ **statistic (total) (N = 145)** Kappa (95%CI)	κ **statistics (males) (N = 81)** Kappa (95%CI)	κ **statistics (females) (N = 64)** Kappa (95%CI)
InterVA versus HCOD	0.32 (0.30-0.38)	0.27 (0.22-0.30)	0.38 (0.32-0.41)
InterVA versus PCVA	0.42 (0.37-0.48)	0.33 (0.30-0.37)	0.52 (0.47-0.54)
PCVA versus HCOD	0.52 (0.48-0.54)	0.47 (0.44-0.50)	0.57 (0.54-0.60)
InterVA + PCVA+ HCOD	0.41 (0.37-0.48)	0.35 (0.32-0.38)	0.48 (0.44-0.52)

InterVA: probabilistic InterVA model, PCVA: physician-certified verbal autopsy, HCOD: hospital cause of death as the gold standard, CI: confidence interval.

**Figure 4:**
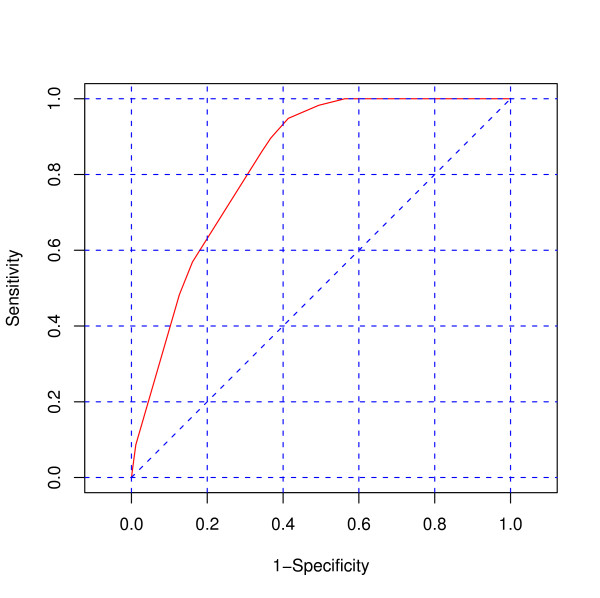
**Receiver operator characteristic (ROC) curve for the InterVA model**. The figure shows the area under the receiver operator characteristic (ROC) curve for InterVA against HCOD. The area under the curve captures the relationship between the sensitivity and specificity of the InterVA method and is therefore indicative of how the method performed with respect to HCOD. The overall diagnostic measure for InterVA model was 0.82, indicating good diagnostic performance of the method. Also, the curve follows the left-hand border and then the top border of the ROC space, indicating an acceptable level of accuracy.

**Figure 5:**
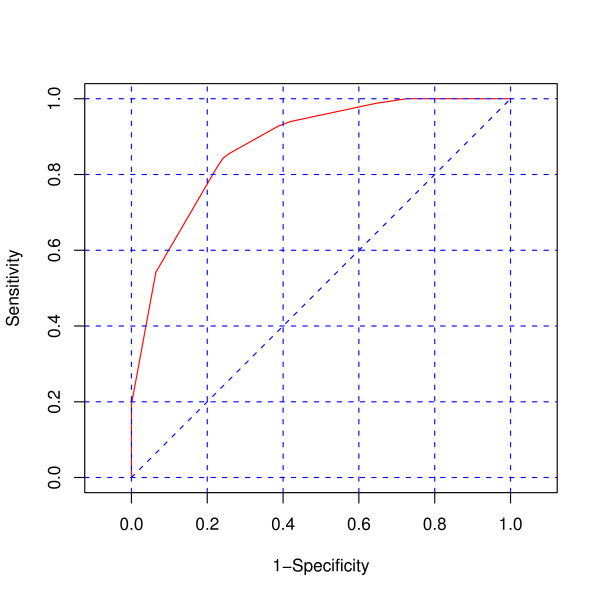
**Receiver operator characteristic (ROC) curve for PCVA**. The figure shows the area under the receiver operator characteristic (ROC) curve for PCVA against HCOD. The area under the curve captures the relationship between the sensitivity and specificity of the PCVA method and is therefore indicative of how the method performed with respect to HCOD. The overall diagnostic measure for PCVA was 0.88, indicating good diagnostic performance of the method. Also, the curve follows the left-hand border and then the top border of the ROC space, indicating an acceptable level of accuracy.

The overall diagnostic performance accuracy of the InterVA model and PCVA are shown in Figures 4 and 5, respectively. The false positive rate (1-specificity) is plotted on the x-axis and the true positive rate (sensitivity) on the y-axis. The area under the curve (AUC) for InterVA (0.82) and PCVA (0.88) were quite good, being close to the ideal value of 1.0.

The results for sensitivities, specificities, PPV, and NPV with their 95% CIs of the InterVA model and PCVA in comparison to HCOD for the five most common causes of death are presented in Table 2. The observed sensitivities and specificities for both methods across the five major causes of death varied from 43% to 100% and 87% to 99%, respectively. The observed sensitivity value for meningitis for both PCVA and the InterVA model was relatively low (43%) as compared to the cut-off value of 60%.

**Table 2:** Validation results for the InterVA model and PCVA against the HCOD in diagnosing the cause of death for the five most common causes of death among 145 adults

Causes of death	Sensitivity (%) (95% CI)	PPV (%) (95% CI)	Specificity (%) (95% CI)	NPV (%) (95% CI)
	**InterVA**			
HIV/AIDS-related death	70 (5-84)	61 (43-76)	87 (79-92)	91 (84-92)
Cardiovascular	52 (34-69)	57 (37-75)	88 (81-94)	86 (78-92)
Tuberculosis (pulmonary)	83 (36-100)	28 (10-54)	91 (85-95)	99 (96-100)
Meningitis	43 (10-82)	33 (8-70)	96 (91-99)	97 (93-99)
Diabetes	63 (25-92)	83 (36-100)	99 (96-100)	98 (94-100)
	**PCVA**			
HIV/AIDS-related death	88 (72-97)	80 (64-92)	94 (88-98)	96 (91-99)
Cardiovascular	70 (51-84)	82 (63-94)	96 (90-99)	91 (85-95)
Tuberculosis (pulmonary)	100 (54-100)	55 (23-83)	96 (92-99)	100 (97-100)
Meningitis	43 (10-82)	60 (15-95)	99 (95-100)	97 (93-99)
Diabetes	100 (63-100)	57 (29-82)	96 (91-99)	100 (97-100)

PCVA: physician-certified verbal autopsy; InterVA model: probabilistic model; NPV: negative predictive value; PPV: positive predictive value; CI: confidence interval

PPV is the number of positives correctly diagnosed through the InterVA model/PCVA (true positives) divided by number of positives diagnosed in hospital (as the gold standard).

NPV is the number of negatives correctly diagnosed through the InterVA model/PCVA (true negatives) divided by number of negatives diagnosed in hospital (as the gold standard).

## Discussion

Although a number of previous studies have been conducted with a view to validating the use of verbal autopsy as a means of determining the cause of death in adults [9-15], to our knowledge this is the first report that has aimed to validate data collected using the new WHO international standard verbal autopsy adult questionnaire against HCOD as the gold standard. The two previous validation studies [5,16] compared the InterVA model against PCVA. We take this process a step further by validating both methods against the standard HCOD to provide data on the performance of both PCVA and the InterVA model.

The model is based on certainty; hence, the effect of causal relationship is difficult to address in our context. Thus, conceptual classification that reflects the real public health issues is as appropriate as is the ICD-10 coding.

Our results are consistent with those of previous studies showing that the InterVA model and PCVA are valid tools to ascertain causes of death [5,16]. The CSMFs obtained were within 5% of the gold standard for four leading causes of death (HIV-related, cardiovascular diseases, meningitis, and diabetes) and were within 8% of the gold standard value for tuberculosis (pulmonary). Misclassification had a greater effect on the reported CSMF estimates (see Additional files 3 and 4). It appears that the misclassification by the model gives a different picture regarding deaths due to HIV and tuberculosis. However, if one considers that tuberculosis and HIV share many clinical features and can occur as a co-infection, a TB/HIV category will show a similar pattern to that derived from the HCOD and PCVA. Similarly, it was observed that for meningitis both the PCVA and the InterVA model misclassified many of the cases to the ambiguous "Others" category. PCVA performed better than the model at an individual level; however, both arrived at broad agreement in identifying cause of death at a population level. For the purpose of mortality tabulation and statistical use, selection of a single condition is required. In some instances, there may be several causes that can be attributed to a death, from which only one cause needs to be identified and selected based on the principle of preventing the primary or UCOD, had there been an effective preventive program [17]. The PCVA inferred stroke to be hypertension, and therefore merging stroke, hypertension, and all heart conditions together in the cardiovascular diseases category was reasonable. Despite Kilifi being one of the poorest districts in Kenya [18], cardiovascular diseases were among the five most common causes of adult death, confirming that deaths from cardiovascular diseases are not restricted to resource-rich communities. Furthermore, one death from sickle cell disease in a 28-year-old patient was correctly classified both by PCVA and by the InterVA model.

Although there are other important causes of adult deaths, our hospital data had two cases of cancer (cancer of the cervix and leukemia), a case of chronic obstructive pulmonary disease (asthma), a case of ischemic heart disease/stroke (stroke cases were due to other underlying causes such hypertension), a case of liver cirrhosis (alcoholic liver disease), a case of renal failure, and two cases of pneumonia. These frequencies were so low that a massive study would be required to meaningfully investigate the performance of the different models for these conditions or subdivisions thereof.

The kappa statistics obtained in the current study (κ = 0.32 for InterVA, κ = 0.52 for PCVA, and κ > 0.40 overall) suggest that PCVA performs better than the InterVA model.

Compared to the gold standard, the diagnostic accuracy of both the InterVA and PCVA were good. The area under the ROC curve is close to the ideal value of one for both methods, suggesting that both methods (InterVA and PCVA) are valid compared to the gold standard. The observed sensitivity values for both PCVA and InterVA model were above 60%, apart from meningitis which scored low sensitivity. This relatively low sensitivity is consistent with a previous study in Kilifi [19] where meningitis yielded a sensitivity of less than 50%. The observed specificity values for both PCVA and InterVA model were good.

Our study had a number of strengths. First, the HCODs were ascertained by experienced physicians with access to a range of high-quality diagnostic facilities. Second, the verbal autopsies were conducted by trained field workers using the new WHO adult verbal autopsy tool. Inadvertently, these results also validate the WHO adult questionnaire. Third, the InterVA model has been shown in several studies to be effective and was also evaluated on a preliminary basis in Vietnam [20] and Ethiopia [5] and found to be good. Overall, the InterVA model and PCVA classified only 4% and 1%, respectively, of all cases in this study as indeterminate, reflecting deaths in which either the respondent was not very familiar with the deceased's illness, there were confusing signs or symptoms, or perhaps there were poor interviewing skills. This percentage is low, and we consider it acceptable given the obtuse nature of the VA process.

Conversely, our study also had a number of limitations. First, it is likely that some causes of death are less likely to occur in a hospital than others, typically those due to accidents, violence, and suicide [21]. As a result, it could be argued that our results might not be generally applicable because of potential differences in the distribution of causes of death in the hospital compared to the community. Second, although postmortem examination is the most accurate way to determine cause of death, such data were unavailable at the Kilifi site. In the absence of such pathology reports, the hospital records were the best alternative. Third, the sample size was small; nevertheless, the overall picture of CSMF for the major causes of death in our study population was similarly determined by both methods. Finally, the absence of some variables in the WHO verbal autopsy adult tool is a factor challenging the accuracy of the InterVA model to be more realistic compared to the gold standard.

## Conclusion

In conclusion, we have shown that both the probabilistic InterVA model and PCVA compared reasonably well with the HCOD in determining the five most common underlying causes of death in a rural community in Kilifi district in Kenya. We hope that our study, albeit small, provides new and useful data that will stimulate further definitive work on methods for interpreting VA data. Inadvertently, this study validated the WHO international standard verbal autopsy adult questionnaire in two ways: first, in collecting VA data successfully for interpretation by PCVA and second, in providing indicators for the InterVA input whose output compared well with HCOD. This study further suggests that both the WHO adult tool and the InterVA model are feasible tools to measure cause-specific mortality, which may potentially inform both health policy and program interventions in resource-limited settings.

## Competing interests

The authors declare that they have no competing interests.

## Authors' contributions

TW conceived the study design and edited the final version of the paper. EB contributed to study design, literature review, interpretation of the results, and drafting of the paper. CN reviewed literature, analyzed and interpreted data, and drafted the paper. GM helped with verbal autopsies data coding/matching, interpretation of the results, and editing of the paper. GN helped with data management aspects and editing of the paper. LM helped in setting up the adult hospital surveillance and editing of the paper. ON contributed in creating hospital data on cause of death. TU contributed in creating hospital data on cause of death. SY contributed in creating hospital data on cause of death. BT helped to conceive the study, established a continuous community awareness and a mechanism for disseminating and implementing the results, and edited the paper. MA designed sensitization messages, implemented a continuous community awareness system, and edited the paper. AE was responsible for managing the adult hospital surveillance and helped in editing the paper. All authors read and approved the final version of the manuscript.

## Supplementary Material

Additional file 1 **Indicators included in the InterVA model but missing from WHO verbal autopsy adult tool**. The majority of missing indicators are disease conditions in adults and variables from the treatment section of the WHO adult questionnaire. Conversely, indicators in the model are not accounted for in the WHO data collection tool. Click here for file

Additional file 2 **Spreadsheet showing cause of death categories assigned by the HCOD, PCVA, and InterVA model**. The spreadsheet shows varying causes of death for each method. These were further categorized into broader cause groups referred to as the "condensed common list" to match each other, especially for causes without direct correlates. Diseases with fewer frequencies were also regrouped; mapping was then done and a common list was generated (collapsed COD list) for easy comparison. Click here for file

Additional file 3 **Pattern of misclassification error: comparison of InterVA model causes of death versus the hospital cause of death**. The table shows patterns of misclassification of cause of death (COD) between InterVA model versus hospital cause of death (HCOD). Misclassification was observed among all COD. Click here for file

Additional file 4 **Pattern of misclassification error: comparison of physician-certified verbal autopsy causes of death versus the hospital cause of death**. The table shows patterns of misclassification of cause of death (COD) between physician-certified verbal autopsy (PCVA) versus hospital diagnosis (HCOD). Misclassification was observed among all COD. Click here for file
