# Supplementary material for: Validating physician-certified verbal autopsy and probabilistic modeling (InterVA) approaches to verbal autopsy interpretation using hospital causes of adult deaths
Source: Popul Health Metr. 2011 Aug 5;9:49. doi: 10.1186/1478-7954-9-49 (PMC3160942; doi:10.1186/1478-7954-9-49)
Supplement: Additional file 1 — Indicators included in the InterVA model but missing from WHO verbal autopsy adult tool. The majority of missing indicators are disease conditions in adults and variables from the treatment section of the WHO adult questionnaire. Conversely, indicators in the model are not accounted for in the WHO data collection tool. [file 1478-7954-9-49-S1.DOC]

**Additional file 1**

| **Indicator** | **Indicator description** |
| --- | --- |
| **disch** | been discharged from hospital very ill |
| **drowsy** | any drowsiness |
| **exc_drink** | any excessive water intake |
| **exc_food** | any excessive food intake |
| **heart_dis** | any diagnosis of heart disease |
| **kidney_dis** | any diagnosis of kidney disease |
| **liver_dis** | any diagnosis of liver disease |
| **Malaria** | any diagnosis of malaria |
| **no_swe_an** | no bilateral swelling of ankle |
| **paral_both** | was there paralysis on both sides |
| **Sickle** | any diagnosis of haemoglobinopathy |

*Data on eleven InterVA indicators were not available in the WHO verbal autopsy tool and so remained null*
