# Supplementary material for: Validating physician-certified verbal autopsy and probabilistic modeling (InterVA) approaches to verbal autopsy interpretation using hospital causes of adult deaths
Source: Popul Health Metr. 2011 Aug 5;9:49. doi: 10.1186/1478-7954-9-49 (PMC3160942; doi:10.1186/1478-7954-9-49)
Supplement: Additional file 3 — Pattern of misclassification error: comparison of InterVA model causes of death versus the hospital cause of death. The table shows patterns of misclassification of cause of death (COD) between InterVA model versus hospital cause of death (HCOD). Misclassification was observed among all COD. [file 1478-7954-9-49-S3.DOC]

|  | **Hospital cause of death (HCOD)** | | | | | |  |
| --- | --- | --- | --- | --- | --- | --- | --- |
| **Cause of death (InterVA COD)** | **Cardiovascular** | **Diabetes** | **HIV/AIDS related death** | **Meningitis** | **Tuberculosis (pulmonary)** | **Others** | **Total (InterVA COD)** |
| **Cardiovascular** | 17 | 1 | 1 | 0 | 0 | 11 | 30 |
| **Diabetes** | 1 | 5 | 0 | 0 | 0 | 0 | 6 |
| **HIV/AIDS related death** | 1 | 0 | 23 | 0 | 1 | 13 | 38 |
| **Meningitis** | 2 | 0 | 3 | 3 | 0 | 1 | 9 |
| **Tuberculosis (pulmonary)** | 2 | 0 | 4 | 1 | 5 | 6 | 18 |
| **Others** | 10 | 2 | 2 | 3 | 0 | 27 | 44 |
| **Total (HCOD)** | **33** | **8** | **33** | **7** | **6** | **58** | **145** |

**Additional file 3**
