# Supplementary material for: Validating physician-certified verbal autopsy and probabilistic modeling (InterVA) approaches to verbal autopsy interpretation using hospital causes of adult deaths
Source: Popul Health Metr. 2011 Aug 5;9:49. doi: 10.1186/1478-7954-9-49 (PMC3160942; doi:10.1186/1478-7954-9-49)
Supplement: Additional file 4 — Pattern of misclassification error: comparison of physician-certified verbal autopsy causes of death versus the hospital cause of death. The table shows patterns of misclassification of cause of death (COD) between physician-certified verbal autopsy (PCVA) versus hospital diagnosis (HCOD). Misclassification was observed among all COD. [file 1478-7954-9-49-S4.DOCX]

**Additional file 4**

|  | **Hospital causes of death (HCOD)** | | | | | | |
| --- | --- | --- | --- | --- | --- | --- | --- |
| **Cause of death (PCVA COD)** | **Cardiovascular** | **Diabetes** | **HIV/AIDS related death** | **Meningitis** | **Tuberculosis (pulmonary)** | **Others** | **Total (PCVA COD)** |
| **Cardiovascular** | 23 | 0 | 0 | 0 | 0 | 5 | 28 |
| **Diabetes** | 3 | 8 | 0 | 0 | 0 | 3 | 14 |
| **HIV/AIDS related death** | 1 | 0 | 29 | 0 | 0 | 6 | 36 |
| **Meningitis** | 2 | 0 | 0 | 3 | 0 | 0 | 5 |
| **Tuberculosis (pulmonary)** | 2 | 0 | 1 | 0 | 6 | 2 | 11 |
| **Others** | 2 | 0 | 3 | 4 | 0 | 42 | 51 |
| **Total (HCOD)** | **33** | **8** | **33** | **7** | **6** | **58** | **145** |

*HCOD: Hospital cause of death; PCVA: Physician certified verbal autopsy ;*
